# American Dental Association

**Published:** 1898-02

**Authors:** 


### Reports of Society Meetings.

### AMERICAN DENTAL ASSOCIATION.

(Continued from page 45.)

*August 6, 1897.—Fourth day.—Morning Session.*

THE meeting was called to order by the President, Dr. Truman, at 10.15 o'clock.

The Secretary read the minutes of the previous session, which were duly approved.

*Dr. Crouse.*—I think the matter of how or whether the transactions of the two societies be published in one volume should be settled.

*Dr. Cushing.*—It seems to me that the proper thing is for each Association to close up its business for itself. There is no reason why the transactions of this meeting should be published in that way; it will make very little difference in the matter of expense, and it will be more simple for the Publication Committees of the two Associations to attend to it individually. But I have a suggestion to make with regard to the transactions of the joint Associations: it would be proper to have that published as an addenda to our transactions and to theirs,—what transpired with regard to the consolidation of the two bodies.

*Dr. Crouse.*—I move that the plan outlined by Dr. Cushing be the sense of this meeting.

Motion carried.

*Dr. Peirce.*—I would move that the present Auditing Committee be instructed to audit all accounts that have been handed in, and that the Treasurer be authorized to settle the accounts as audited.

Motion carried.

### DISCUSSION OF DR. CROUSE'S PAPER.

*Dr. Patterson.*—Dr. Crouse says that the amalgams upon the market do not exhibit the same peculiarities upon different mixings. I just want to make the statement that it is my opinion, and I think it is the correct one, that those amalgams will exhibit the same tests if they have been uniformly treated, and that is where the trouble lies. Dr. Crouse, so far as he is before the Association, does not commence on a basis by which his experiments are of any value, to my mind. So long as those mixings are made with different amounts of mercury and amalgam, they will forever be different. He must use the positive amount of amalgam and mercury, which is the proper amount to be used, before these experiments will be of any value. So long as we cannot reach that point, what is the use of taking different materials mixed by different people and testing them? It proves only what one man does, and is not of any scientific value. When Dr. Crouse says that Dr. Black was the first one to make these experiments with the micrometer and other instruments, he is certainly mistaken, because history teaches us that many years ago Thomas Fletcher, of Warrington, England, made most of these important experiments on the same lines. I am very much interested in amalgam, and very much interested in having some settled and definite way of treating the various makes of amalgams which shall produce a definite result. It is true that all the amalgams on the market require a definite amount of mercury, but let each one be treated on a basis. By a perfect amalgam I mean one that will make a mass which will be a perfect, harmonious filling; that each atom will be satisfied with mercury and no excess used, because when an excess is used the whole mass is jeopardized. I know that some of the amalgams are very carefully tested, and every portion of the ingot is absolutely tested. It may not be so with all, but I have found that the manufacturers of the different amalgams make them very uniform, and when they are uniform and treated scientifically from a basis the results will be uniform. I have told Dr. Crouse privately, and I say it now, that I will mix not only twelve blocks, but twelve times twelve blocks, and let him test them with his instruments, and I am sure the results will be the same. I would have done so, but the instruments with which I work are not here.

*Dr. Taft.*—There are so many phases of this subject that it is hard to know what to present in reference to the matter. These experiments are very interesting, and it is to be hoped that from these and like experiments something more will be derived than has yet been found by anybody. I have not gone very extensively into minute experimentation with this material, but there are facts that I know about it. One mistake is the variation in the behavior of all such alloys, especially those into which mercury enters, and even the alloys of other metals. As you mix two or more metals together their behavior is different. Many times we choose the direction, and we look only in that direction. We may all have predilections which lead us to look in those certain directions, and we are more apt to find things than when we do not. I have found that the different amalgams will vary very much; but the same alloy mixed with mercury,—and pure, distilled mercury it ought to be,—manipulated as nearly as they can be manipulated, will give the same results. I have seen amalgam giving apparently good results when placed in the mouth, and have seen the same amalgam utterly fail. It may be said, however, that this failure is dependent upon the mode of manipulation or the various environments to which it is subjected in different mouths. Some mouths are clean and healthy, and others are not; some have good secretions in the mouth, others have not; and those things certainly affect the material. The mouth is a severe test for anything that is liable to undergo decomposition. The fluids of the mouth are exceedingly complicated in character. They are subjected to variation from one state to another, and on this account whatever is placed in the mouth is subjected to a very active influence, so far as decomposition is concerned. In some mouths a standard amalgam, for instance, may be placed, and serve the purpose for which it is employed. The same material in another mouth will show oxidation, and in others contraction. It is this variation in behavior that constitutes one of its great objections to me. If I could find one that is uniform I should be more inclined to favor it. The matter of manipulation is, of course, a point upon which a great deal hinges. That was illustrated in the use of oxyphosphate. Discussion has been had upon the conditions of oxyphosphates under the margin of the gum. In many cases I have seen it stand just as well under the margin of the gum as anywhere else, dependent, I presume, on the manner in which it was manipulated. I know just as well that in many other cases it fails in a very little while.

These are my objections: The liability to shrinkage, the liability to change of form, the liability to oxidation or decomposition. We find that the materials unite with other substances and blacken the mouth. There is another point with regard to solidification: take some varieties of amalgam and break it open, and you will find a good deal of crystallization has been effected. In other cases this crystallization will have almost entirely failed, and the mass in its solidification has assumed a granular form, which is very different from the form where perfect crystallization takes place. My attention was drawn to this when I experimented with crystal gold. I found the same gold, manipulated in the same way, would act differently. Sometimes perfect crystals would form, sometimes small crystals, and sometimes large ones, and in other cases not any crystals at all, but a granular mass that had no more cohesion than particles of sand would have. In the crystallization, when perfectly made, there would be strong cohesion; in the other cases the mass would fall apart like so many beans in a pan. I think the environment at the time of the manipulation influenced the results. Sometimes the atmosphere affected it, and it was impossible to get a perfect crystallization. On a clear, cool day, good results would be obtained; and on a dark, lowering day it would be almost impossible to get crystallization. I think the electrical condition that may exist in the atmosphere at the time of the operation has a great deal to do with it. There are so many circumstances adverse to the results that we all desire that I have yet to use the first amalgam in the mouth. My experiments of forty years ago led me to this point, and I could not conscientiously use it. I might have saved a great many teeth with it, but had I used it there would have been a great many more failures than I have had with better materials. I advise against anything that I cannot conscientiously employ myself.

*Dr. Custer.*—The slight oxidation of an amalgam filling is the best thing that can happen to it, not to the degree of blackness, but a slight tarnish. That is the same as polarization which takes place in a cell, and galvanic action is stopped; so I believe oxidation is one of the best things that can happen to an amalgam filling.

*Dr. Peirce.*—It is a law of nature that similar things, being similarly acted upon, must bring a similar result. The question comes, when we arrive at a definite conclusion regarding the amount of alloy and the amount of mercury that is best adapted to make an amalgam, Shall we not in future have those placed in capsules, to be used together, so we get different mixtures? The amount of trituration varies also. Having an equal amount of alloy and mercury, you want a uniform amount of trituration to produce the same results. Then, too, we place it in different environments, which must also have an influence on its condition. In some mouths it hardens at once; that is due to environment. If we can come down to the facts in each case, and insure a uniform action in every particular, then we shall always have uniform results, but on account of the environment in the mouth we cannot do this. We can approach it by having the proper amount of alloy and mercury, and then we can approximate the proper amount of trituration for the preparation of the plastic filling. As we approximate it we bring a better result, but it is uncertain, because we never can tell the conditions of the environment under which it crystallizes or hardens, and we cannot control that.

*Dr. Crouse.*—I think that some of Dr. Patterson's propositions are correct. As to the experiments of Fletcher, I remember reading them, but not as I should read them now; I shall take them up again and read them over. We want to settle on what combination of metals, when treated properly, will stay as you put them in any ordinary atmosphere, and be the strongest, and not shrink or expand. Then there is another question that the profession must settle. It is not possible to teach every dentist how to make his own amalgam,—how to manipulate it. It is not settled by those who have made the most accurate tests whether squeezing the surplus of mercury out of an alloy is better or not. The strongest blocks I have made thus far are amalgams that would mix and pack without squeezing out surplus mercury, and yet I have made some good ones by taking a towel or chamois and wringing it out dry. I think the alloy that will be the most useful will be one that is settled by definite experiment,—one which will be the strongest and flow the least, and have the mercury in one capsule and the amalgam in another, and then be mixed together. I have made too few tests of the blocks we had here. We put one in, and it would not stand a stress of fifty pounds, while another stood four hundred pounds. They were made of the same alloy. If there is that much variation in the manipulation, in addition to the variations in the alloys and the environment, is it any wonder that the careful observer has often become disgusted with amalgam fillings? Dr. Patterson says he has found the manufactured alloys run uniform. He has been more fortunate than I have been, or he has not made his accurate tests as I have, and I should question that statement on that account. You hardly ever find a make where two preparations under the same label run alike, where they put up one hundred ounces at one time and one hundred ounces at another time. I could name a combination which the burning out of one ingredient in one hundred will alter entirely. That does not come down to the test that will keep micro-organisms out from between the teeth. I believe that that will be the proper way to put alloy on the market with the mercury. It would be safe to say that from an alloy that would make the mass as plastic as could be handled well, and will hold as much mercury that you cannot squeeze out the surplus, you will get the best results.

Subject passed.

*Dr. Ottofy.*—I move that at the final adjournment all the permanent members present sign their names in a book, which we have here, and that H. A. Smith and Jonathan Taft, who were present at the opening of the organization, sign at the end.

Motion carried.

*Dr. Crouse.*—I move a vote of thanks to the proprietor of this hotel for the kindly treatment of the Association during our stay.

Motion carried.

*Dr. Taft.*—It was mentioned that but two of those present were at the organization of this body. A number of those who were present at that time have passed away; others still remain, but are not with us to-day, and when we look back to that occasion it produces a feeling of sadness that the career of a body that has accomplished so much as this one now terminates by the inevitable trend and run of progress and of circumstances in the profession. To those who have been long years with it, and especially to those who were at the organization of it, it produces a feeling of sadness that associations which we have had here together as members of this body will now cease forever in the capacity in which they existed heretofore. It is not unreasonable that these feelings should be indulged in; they cannot be avoided, by some of us at least. This Association accomplished a grand work for the profession, not only in this country, but wherever dentistry is known on this wide earth. It has ever striven for the betterment, the improvement, and the elevation of the profession, and when we compare the condition of the profession at the origin of this body with what we see now, we can fully realize that fact. The profession stands to-day in a vastly advanced position, and it is largely due (although not entirely) to the work that has been done in and by this Association. We all know what it has done for the stimulation of the work in State and local societies, and for the literature of our profession and our educational systems. It has fostered all these things, and they are due to the efforts of this Association. It is to be hoped that the memory of this body will not pass from any who have been in its ranks and co-operated in the work which it has so nobly done; that its career will be held sacred in the memory of all its members. It has steered well through the difficulties which it has encountered, and it has come out to a grand end, and that into which it has merged will go on and be as efficient, as honorable, and as well directed in all the future years as this body has been and done in the past. May we all realize this fact in years to come!

*Dr. Corydon-Palmer.*—I can but have a feeling of sadness when I remember the long years that we have passed through, and how this organization was first commenced. I was present at the time. As I have been a member and have gone along with it all those years, I feel sad at the thought that it will soon pass away.

*Dr. McManus* (of Hartford, Conn.).—Two years ago, in New England, two societies, one thirty-two years in existence and the other thirty-three years, came together and voted to form a joint society. One was the New England and the other the Connecticut Valley. I was a member of the Connecticut Valley from the first year. A few of us felt sad, as Dr. Taft and Dr. Palmer feel to-day, at the disbanding of the societies, but it was the vote of the majority that it was the best thing to do to form a new association, called the Northeastern Association. It was a movement in the direction of trying to strengthen the societies, and still have an organization where the different members could meet together and keep up their friendly relations, and to work up an interest in the State societies. Many of us did not think at that time that what has been accomplished here would be done. We believed that the American Dental Association would go on, and that the Southern Dental Association would also continue. In the movement that is taking place now, I have been gradually brought to see that it was the proper thing to do, and I do not feel as badly as I did when we changed our society up in New England. It is over thirty years since Dr. Taft came up there to awaken interest in that society, and many of us remember with pleasure his visit to Connecticut with Dr. Atkinson. Their presence on that occasion stimulated the dentists of the region. Our State organization had been in existence, but the presence of those two gentlemen gave renewed vigor to it. New England has always been very grateful for the visit of Dr. Taft at that time. This movement has been very successfully accomplished, and I have great reason to hope that the National Dental Association will do even greater work than the American Dental Association has done in the past; but if it only goes on in the same way, if it will do as much as the American Dental Association has done in the past, it will have glory enough.

The American Dental Association has been exceedingly kind to me. As far back as 1865 the members honored me with the vice-presidency, and some years later they again elected me vice-president. I felt I have had enough honor, but yesterday they again elected me vice-president, and I must thank all the members of the

American Dental Association. They did so because they had a kindly feeling for me, as I had been a member so long.

*Dr. Taft.*—The visit that was made throughout the East and other parts of the country, as referred to by Dr. McManus, was the work of this body. It was not Dr. Atkinson's work nor mine: we were sent forth by this body to do certain work. The committee did as well as it could, and if any good was accomplished it is a matter of great gratification to me. I wish to say that I disclaim any credit for that work at all; the credit of it belongs to this body. It originated when this body took a fostering care of association work all over the country.

*Dr. H. A. Smith.*—I have been a society man from the beginning. On the day I received my degree I joined the old Mississippi Valley Association, and was a member until its dissolution. I often said if my relations with dental associations had been cut out of my dental life it would have been comparatively a blank, nothing but drudgery and hard work; but it has been the meeting with my fellows that has stimulated me. I came into this society as a young man, and have been in close attendance ever since. To me it has been almost invaluable. The members of the dental profession are the best fellows alive. They are cultured to the highest degree, they are sociable, and I never enjoy myself so much as when I am with them. Then the professional side of it. Think of meeting men who have reached the highest attainments in dentistry and given their best experience! From that stand-point it has been of immense value to me. I feel many regrets that it goes out of existence, yet I think it will go on in the new body, and we will go to new efforts in every way.

*Dr. Crouse.*—I remember very well my first meeting in the American Dental Association. I was about seventeen years old, and thought I was a dentist. I attended a course of lectures and went into partnership with a man I studied with. A call came to that office, signed by Dr. Cushing and some other gentlemen, to form a State society. The American Dental Association met that year in Chicago. There were some entertainments to be given, and the delegates were asked to come up and give their tickets, and I went up with the rest. I remember seeing Dr. Taft, Dr. Atkinson, and some one else whose name I have never been able to get. I remember just how he looked, and I remember he built out the corner of a front tooth, which was a marvel to me at that time. I went home and told my brother I was not fit to fill teeth according to the way they filled teeth there. Sometimes a young man needs encouragement, and my brother gave me that. At the Centennial in Philadelphia, somebody put me on the Executive Committee, and I have been on ever since, except one year when I resigned. If you want to enjoy a society, get in and work. The fellows who do not do anything do not get the good of it. It is the men who work and do something. I would be a miserable being if I did not have something to do. That would be the plea that I would make,—have all the members do something for the society. To me, what this society has done cannot be estimated or spoken of in words. To this and my State society I owe everything that I have made of myself.

*Dr. Peirce.*—I want to say a word to bring to memory a friend and brother from Philadelphia, Dr. J. H. McQuillan, who probably was as instrumental as any one man in forming this Association. I remember several conversations with him, and the deep interest he took in it. I remember he was at Washington and at the subsequent meeting at Niagara Falls. I was secretary at the convention that year, and was not at the organization. To Dr. McQuillan in its organization, to Dr. M. S. Dean in the carrying on and working out of the society, we owe much. He brought me into it, and everything I gained the first few years I gained from him. He was not a fluent talker, nor did he often take the floor; but he was a good worker. He influenced me more than any other man except Dr. McQuillan. The influence of the organization on the dental profession we cannot estimate. It has been unlimited in the education and stimulation of young men. I can remember the first paper I ever brought before the Association, and the kindness with which it was received and the stimulation it was to me to do other work. What has been the influence on myself, I think was the influence on many others,—to do original work and formulate their ideas, and bring them to an association where they were fully appreciated. In that way we cannot over-estimate the influence this organization has exerted on the dental profession in this country.

*Dr. Fillebrown.*—I feel as much interest in this society as any one who was not an original member, and not a constant attendant until recently. I feel with Dr. Crouse that my greatest happiness has been since I became a worker in it. To be a worker is the only way to enjoy a society. You know a grain never produces the fruit until the seed dies. Progress is built on the ruins of the past; so all through history we find that a race has done its work and faded away, because their work was done. The world goes on, and new circumstances come that make it impossible for the former association to do the work demanded of it. The American Dental Association has arrived at the point that many other associations have in the past. There was a work to be done, which under the circumstances the American Dental Association, as such, could not do. The only way for it to be done was for a new organization to be formed. Happily that has been consummated. Our good friend Dr. Smith says the spirit of the Association passes into the new organization. I would improve upon that by saying not only the spirit, but the spirits. Each individual spirit goes into that new association to give life to it, and join with it the wandering spirits that have been floating around unorganized and not in harmony with it. There is the happy feature that by the giving up of this organization the same ambitions, enthusiasm, and scientific attainments join hands all over the country, and come together in one strong union that shall carry it on to greater perfection. May the new association long live, and always look back to the American Dental Association as the basis which has made it possible that it should exist.

*Dr. Taft.*—I trust it will come into somebody's mind to prepare a condensed history of this society, and publish it, embracing those who have been the leading actors in its work, especially those who have passed away, and embracing the principal acts and the leading work which this society has done. Of course, this history can be gathered up from its publications; but it would be well to have it in a few pages.

*Dr. Patterson.*—Some fifteen years ago, when I came as a delegate from the Kansas association to the meeting at Niagara, what impressed me then, and has since impressed me, was the cordiality with which I was received. The gentleman who took my fee said, "Ah! all the way from Kansas; glad to see you." Dr. Taft and Dr. Kingsley, Dr. Barrett and Dr. Rehwinkel I also remember. I had lived thirty years and had never seen the ocean. I always remember the first meeting I attended, along with that fact. Since that time, the kindness I received has been more than I deserve, and I have tried in my weak way to make some return for the extreme kindness which I did not expect and for which I was not fitted. The memory of the American Dental Association, it seems to me, is safe in the hearts of its members, and the work it has done will not be forgotten.

*Dr. Cushing.*—I cannot let this occasion go by without saying a word. For nearly twenty years, through the partiality of the

Association, I have sat at the secretary's desk, and I have seen the members as perhaps nobody else has seen them, constantly before me as they come and go. I recall the old friends who have gone from us, and miss them every year as I take my place here. I have missed them constantly, and shall continue to miss them. I am not in a mood to make a speech, even if I were able to do so. It seems to me we are very much in the position that we would be if we were standing at the death-bed of a dear friend, whom we feel confident has gone to a higher life.

*Dr. Taft.*—I move that the thanks of this Association be tendered to the officers of the Association, and especially to our worthy secretary, Dr. Cushing, who has served this body so long and so faithfully, and to whom great credit is due for the work he has accomplished in this direction.

Unanimously carried.

The secretary then read the closing minutes, which were approved.

*Dr. Truman.*—Members of the American Dental Association. Since Dr. Allport, a warm personal friend of mine, began as president of this Association, up to this period, when I am called upon to close it, I cannot conceive that I have gone through any more trying occasion than this. When I left my home to come to this meeting, it was with a joyous feeling that we would unite with our Southern brethren, and form a new organization, and that has been accomplished. I only regret that in that organization those who had it in charge failed to read the signs of the future. I do not propose to criticise the proceedings here. I say it is with profound regret that they could not see their way clear to making some arrangement by which scientific work could receive financial support and should be made to live independent of daily bread. I regret that they did not see their way clear to eliminate, to my mind, the worst feature in associated efforts,—permanent membership; but I look forward hopefully to the time when that will be accomplished. I have one more regret,—that this American Dental Association should be left to die as it is dying to-day. To my mind it should have lived, and there was no reason why it should not have continued. It could have been made a branch. You have now before you the necessity of forming a new organization in the Eastern part of this continent.

We bring our wreaths and lay them over our dead. We gather at the tombs of our loved ones and take pleasure in recalling their virtues and the work they have performed. It is well that we have met here to-day, not only to mourn the loss of this Association, but to recall to memory the work it has performed for dentistry in this country and the world. As we pass away from this hall, sadly remembering that we shall no more meet together under its banner, may we return to our homes feeling that the future will take care of itself; that the profession of dentistry is worthy of our best efforts, and that we will, as long as we may live, support any organization that may be established, hopefully looking forward to greater improvement.

In closing, it is, perhaps, unnecessary for me to say to you, members of the American Dental Association, that no more painful duty has been given me to perform than this of closing the sessions of this body. I have been an associate in its labors for many years, and have felt that it has stimulated me as it has others, and now the last word must be said, and in my official capacity I declare from this platform that the American Dental Association has completed its work and is disbanded forever.

---

